# Deep Learning Approach for Imputation of Missing Values in Actigraphy Data: Algorithm Development Study

**DOI:** 10.2196/16113

**Published:** 2020-07-23

**Authors:** Jong-Hwan Jang, Junggu Choi, Hyun Woong Roh, Sang Joon Son, Chang Hyung Hong, Eun Young Kim, Tae Young Kim, Dukyong Yoon

**Affiliations:** 1 Department of Biomedical Informatics School of Medicine Ajou University Suwon, Gyeonggi-do Republic of Korea; 2 Department of Brain Science School of Medicine Ajou University Suwon, Gyeonggi-do Republic of Korea; 3 Department of Psychiatry School of Medicine Ajou University Suwon, Gyeonggi-do Republic of Korea; 4 Department of Biomedical Sciences Graduate School of Medicine Ajou University Suwon, Gyeonggi-do Republic of Korea

**Keywords:** accelerometer, actigraphy, imputation, autoencoder, deep learning

## Abstract

**Background:**

Data collected by an actigraphy device worn on the wrist or waist can provide objective measurements for studies related to physical activity; however, some data may contain intervals where values are missing. In previous studies, statistical methods have been applied to impute missing values on the basis of statistical assumptions. Deep learning algorithms, however, can learn features from the data without any such assumptions and may outperform previous approaches in imputation tasks.

**Objective:**

The aim of this study was to impute missing values in data using a deep learning approach.

**Methods:**

To develop an imputation model for missing values in accelerometer-based actigraphy data, a denoising convolutional autoencoder was adopted. We trained and tested our deep learning–based imputation model with the National Health and Nutrition Examination Survey data set and validated it with the external Korea National Health and Nutrition Examination Survey and the Korean Chronic Cerebrovascular Disease Oriented Biobank data sets which consist of daily records measuring activity counts. The partial root mean square error and partial mean absolute error of the imputed intervals (partial RMSE and partial MAE, respectively) were calculated using our deep learning–based imputation model (zero-inflated denoising convolutional autoencoder) as well as using other approaches (mean imputation, zero-inflated Poisson regression, and Bayesian regression).

**Results:**

The zero-inflated denoising convolutional autoencoder exhibited a partial RMSE of 839.3 counts and partial MAE of 431.1 counts, whereas mean imputation achieved a partial RMSE of 1053.2 counts and partial MAE of 545.4 counts, the zero-inflated Poisson regression model achieved a partial RMSE of 1255.6 counts and partial MAE of 508.6 counts, and Bayesian regression achieved a partial RMSE of 924.5 counts and partial MAE of 605.8 counts.

**Conclusions:**

Our deep learning–based imputation model performed better than the other methods when imputing missing values in actigraphy data.

## Introduction

An accelerometer-based actigraphy device can measure the movement of the person wearing the device by capturing acceleration in a single axis or in multiple axes of motion. By mounting an actigraphy device on the wrist, ankle, or waist, researchers or physicians can measure the amount of movement or movement patterns. One study [1] has used this tool to detect changes in activity resulting from neurological diseases, including Parkinson disease, depression, dementia, and affective disorder. In addition to its utility for certain diseases, this device also has been used in the analysis of sleeping patterns or activity recognition (such as running, walking, or sitting) in healthy participants [2,3]. Furthermore, because accelerometer-based data are objective whereas self-reported data are subjective and can be biased by participant recall, accelerometers and actigraphy devices have increasingly been used in clinical trials to collect real-world data [4,5].

Because data recorded over a few days to a few months are usually required to be able to analyze activity patterns, studies have frequently faced the issue of participant adherence to use [6]. Many indices of activity such as interdaily stability, intradaily variability, or relative amplitude require full records over a day, so even a small number of missing values hinders the use of the records captured on that day [7]. In one previous study, the researchers collected 17,542 day-long activity records, but only 2003 (11.41%) records could be used in their study because of missing data [6].

To overcome this issue, statistical models have been suggested to impute missing data [8,9]; however, these models require assumptions about the distribution of the missing values (such as missing at random, missing completely at random, or not missing at random assumptions) and using a specific assumption may not be appropriate for a given study.

Recent studies [10-12] have confirmed that deep learning models which are able to learn features from raw input without manual feature engineering or statistical assumptions perform well in many tasks, including data denoising, detecting and classifying objects in images, and predicting values. An autoencoder is a deep-learning method that can extract key features from input data and restore them; therefore, an autoencoder can also be used for reconstructing original data from corrupted data [13]. This approach has been used to impute missing values in traffic data, which are similar to actigraphy data—both data sets have time-series characteristics [14]. Furthermore, another study [15] showed that the autoencoder-based approach can extract core features from activity data. These findings suggested the possibility of improving the performance of imputation in activity data using a deep-learning model. In contrast to statistical approaches, the deep-learning method can discover hidden patterns from the data themselves without any specific assumptions about the missing values. Assuming that missing values could be treated as a type of noise in the data, we developed an autoencoder-based model to restore missing values in actigraphy data. We used three actigraphy data sets and compared imputed data from our model with those of other methods.

## Methods

### Ethics

This study was approved by the Ajou University Hospital institutional review board (AJIRB-MED-EXP-17-470). Before collecting actigraphy data from patients at Ajou University Hospital, we received their informed consent (Korean Chronic Cerebrovascular Disease Oriented Biobank, KCCDB). The other two data sets used in this study, the National Health and Nutrition Examination Survey (NHANES) [16,17] and Korea National Health and Nutrition Examination Survey (KNHANES) [18-21], are publicly available. All data were deidentified and used only for retrospective study.

### Overview

**Figure 1:**
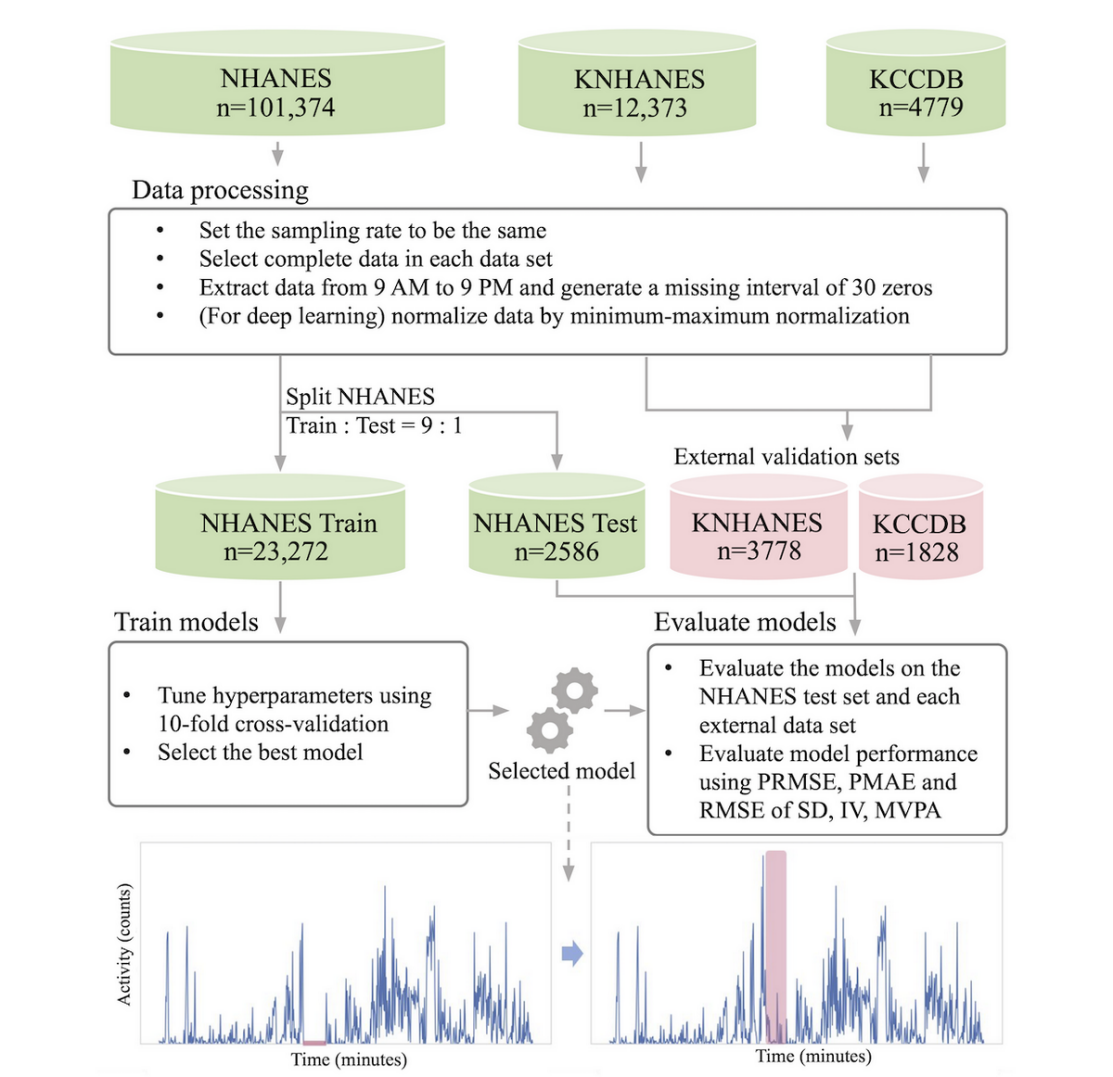
Overview of the study and data where n indicates number of records (days). IV: intradaily variability; KCCDB: Korean Chronic Cerebrovascular Disease Oriented Biobank; KNHANES: Korea National Health and Nutrition Examination Survey; MVPA: moderate-to-vigorous physical activity; NHANES: National Health and Nutrition Examination Survey; PRMSE: partial root mean square error; PMAE: partial mean absolute error; RMSE: root mean square error.

This study consisted of four phases. First, we collected activity data from three different actigraphy data sets. Second, complete data for each day were selected from the data sets and preprocessed to generate artificially corrupted data. Third, models were constructed from the data, including our deep learning–based imputation model. Finally, models were evaluated with performance indices. Details of the procedures are shown in Figure 1.

### Data Sources

Three actigraphy data sets were used for this study—NHANES, accelerometer-based actigraphy data collected over four years (2003-2006) from 14,482 individuals living in the United States; KNHANES, accelerometer-based actigraphy data collected over two years (2014-2015) from 1768 people living in South Korea; and KCCDB, accelerometer-based actigraphy data were collected over two years (2014-2015) from 177 patients who had visited Ajou University Hospital for evaluation or treatment of cerebrovascular disease.

The NHANES data set was collected using a uniaxial accelerometer-based actigraphy device (ActiGraph AM-7164, ActiGraph LLC) which gathered only z-axis data. The KNHANES data set, though collected using a triaxial accelerometer-based actigraphy device (ActiGraph GTX3+, ActiGraph LLC), also consisted of only z-axis data (only these data were made available to the public). In contrast, the KCCDB data set was collected using a triaxial accelerometer-based actigraphy device (Fitmeter, Fit.Life Inc). The triaxial data were aggregated into a single magnitude vector using the formula 
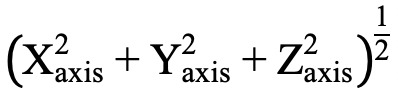
 (Multimedia Appendix 1). In this study, data from each device are used in their original format and values from one device are not comparable to those from others since activity units, device type, and device range (±g) vary [22]. We developed the autoencoder-based imputation model with 90% of the NHANES data set (the training data set); the performance of the model was evaluated using the other 10% of the NHANES data set (test data set) and was externally validated using the KNHANES data set. The KCCDB data set was used to test whether the model, trained with uniaxial data, could also be used with triaxial data.

### Data Processing

First, because the KCCDB data set has a higher sampling rate (0.1 Hz) than those of the other data sets (0.016 Hz), the KCCDB data were downsampled by averaging the values for each minute. As a result, each record consisted of 1440 values per day.

**Figure 2:**
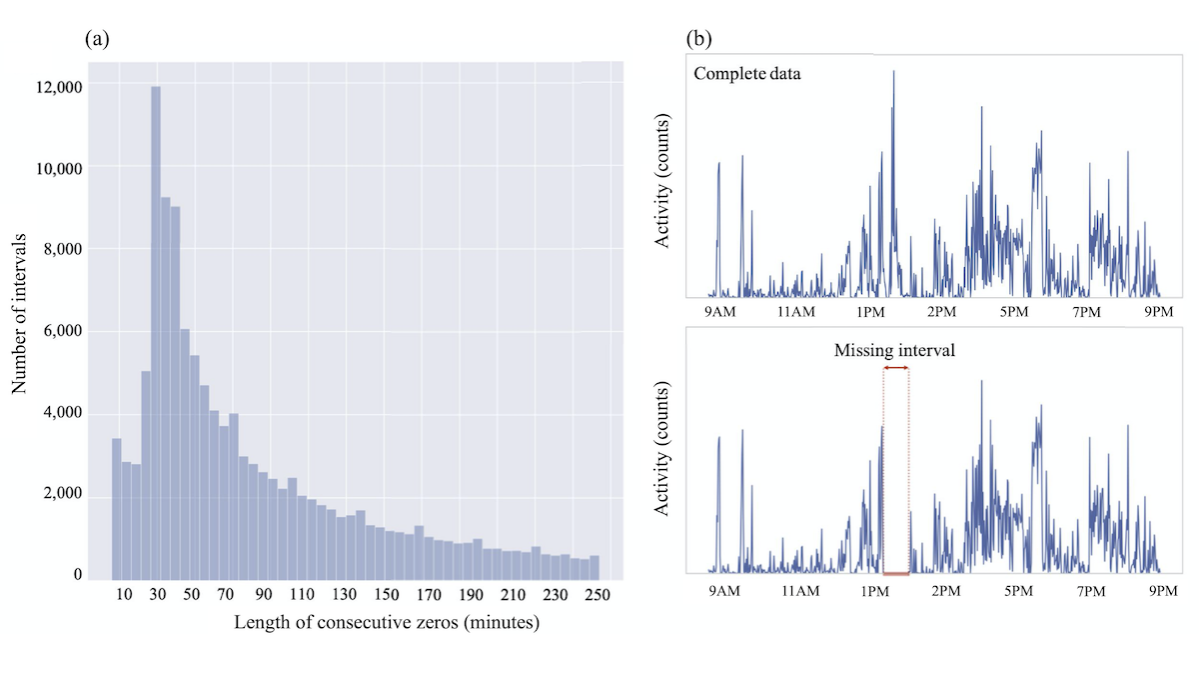
(a) Frequency of missing data intervals found in the NHANES data set. The interval of approximately 30 minutes occurred most frequently. (b) Example of a complete data record and of a record with missing data interval.

In previous studies, a missing interval has been defined as a period (of 20, 30, or 60 minutes, depending on the study) over which zero values are continuously repeated [23-25]. For this study, we defined a missing interval to be 30 minutes or more of consecutive zeros. We found that intervals of such lengths occurred most frequently in the NHANES data set (Figure 2). We found the frequency of missing intervals less than 30 minutes was the highest in both datasets (Multimedia Appendix 2). Only complete records from the data sets were used so that we could calculate the reconstruction error for the missing intervals by comparing the imputed values with the original values.

Finally, we extracted the data from 9 AM to 9 PM, which is the most active period for humans [23-25], and overwrote a random 30-minute interval with zeros in each record. Consequently, each record used in the study had 720 values (over the 12-hour period from 9 AM to 9 PM), in which 30 consecutive zero values were included as a missing interval. Moreover, we conducted additional experiments with 90 and 180 consecutive zero values to confirm the imputation robustness for longer missing intervals. Preprocessing algorithms were written in Python (version 3.6; scikit-learn).

### Models for Imputation

#### Mean Imputation

Mean imputation is a method of replacing a missing value with the mean value from the other instances of valid data at that time [26]. For example, if there is a value missing at 12:30 PM in a certain record, the missing value is replaced with the mean of the values from 12:30 PM in the other records. Mean imputation, algorithms were written in Python.

#### Zero-Inflated Poisson Regression Model

To compensate for the disadvantage of a single imputation method—where missing values are replaced with a single value—the multiple imputation method generates several data sets and the results are combined into a single result to replace the missing values. In this study, the multiple imputation by chained equation approach (also called fully conditional specification) was used for multiple imputation. (1) All missing values of each variable were filled using the mean imputation method. (2) The regression equation, in which the dependent variable is the variable to be imputed and the independent variables are the other variables surrounding the dependent variables, was developed. Then, missing values were replaced by a value estimated by the regression equation [27]. After changing the dependent and independent variables, the process was repeated. The zero-inflated Poisson model has previously been used as a regression model for activity data in which a large portion of the data had zero values [28]; similar to the previous study, the lead and lag options were added to reflect the values of the activity before and after the missing variable. In this study, we applied the zero-inflated Poisson regression model using R (3.3.1) and a package (accelmissing) developed in a previous study [28,29].

#### Bayesian Linear Regression Model

The process was the same as the zero-inflated Poisson model, but Bayesian multiple imputation utilized Bayesian linear regression. Because the Bayesian model aims to find the parameter for the posterior distribution and take a sample from the estimated distribution, the imputed values of this approach can be negative, which cannot exist for the units defined for these devices. Hence, the negative values were replaced with zero values. Bayesian regression algorithms were written in Python.

#### Zero-Inflated Denoising Convolutional Autoencoder (New Method)

An autoencoder is an unsupervised deep-learning method. Its aim is to make its output *X'* approximate the input *X*. An autoencoder consists of an encoder that compresses the information of the input into a compressed-information (or hidden state) *Z* and a decoder that restores *Z* back to *X* as closely as possible:


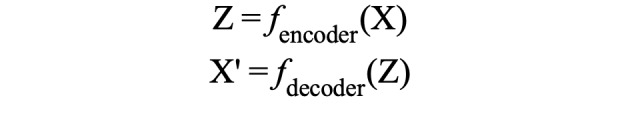


Because *Z* has fewer dimensions than the input, and because the decoder must restore a value close to the input value by utilizing the information in *Z*, the autoencoder must learn the key information of the input in the hidden state in order to reduce the reconstruction error.

A denoising autoencoder is a type of autoencoder that restores noisy input *X_noised_*, which contains masking noise, to the denoised output *X'* [30]. Here, noise refers to data that distort the original data. The zero values were treated as noise distorting the data in this study.

A convolutional neural network is a deep-learning method commonly adopted for analyzing images [31]. A convolutional neural network can extract the information from a specific area of the data by applying filters to the input data. This approach compensates for the disadvantage—that of being unable to utilize positional information—found in simple neural networks. One-dimensional sequential data can be analyzed using a one-dimensional convolutional neural network; studies on this approach have been actively conducted in various fields such as voice synthesis or biosignal analysis [32,33].

**Figure 3:**
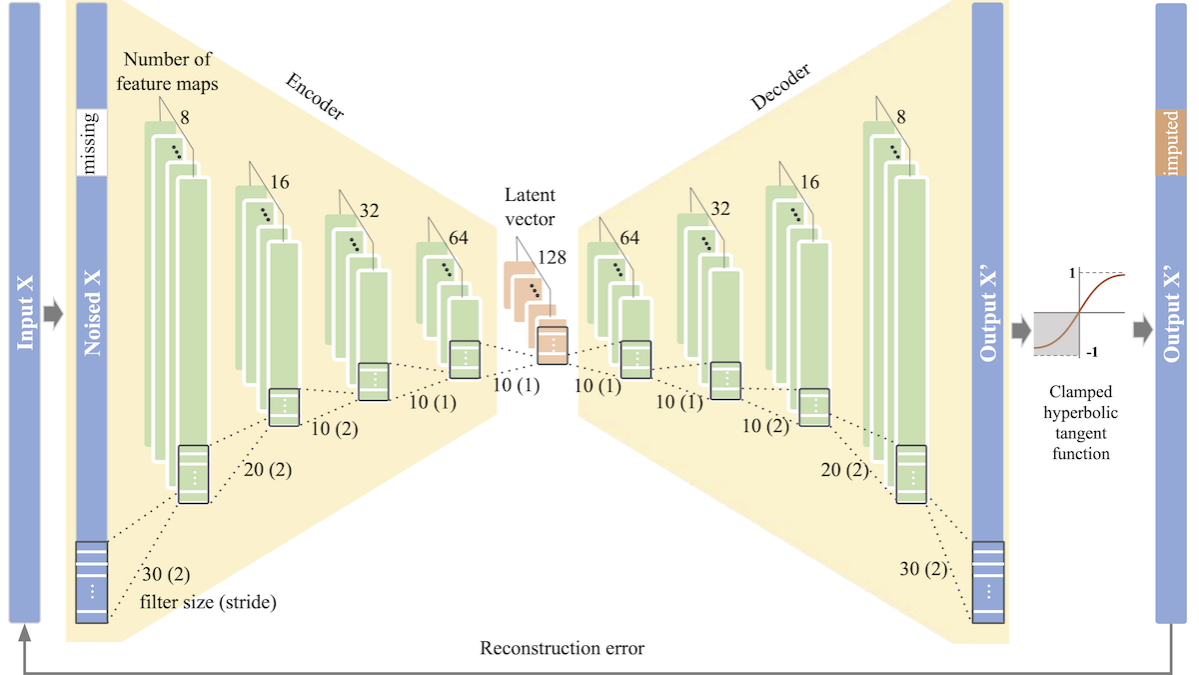
Model architecture for zero-inflated denoising convolutional autoencoder consisting of encoder with 5 convolutional layers in which the filter size and stride decrease and the number of feature maps increases and decoder with 5 transconvolutional layers in which hyperparameters are symmetrically the same as those used in the encoder.

Our model, the zero-inflated denoising convolutional autoencoder, consisted of an autoencoder that encoded and decoded the data using a convolutional neural network and a unique activation function designed for the zero distribution at the last layer. The zero-inflated denoising convolutional autoencoder received corrupted data as input, then compressed and recovered these data using a convolutional autoencoder, as shown in Figure 3.

We used the PyTorch (version 1.4.1) framework to construct the deep learning model [34]. Input data for zero-inflated denoising convolutional autoencoder were normalized (minimum-to-maximum normalization); then, each zero in the missing interval was replaced with a value of 0.5. As the input data were encoded, the filters of each convolutional neural network layer in the encoder compressed the regional information into a feature map. The size of the feature map decreased and the number of feature maps increased every time data passed through the convolutional neural network layer so that latent vectors with various perspectives could be extracted. After the corrupted data had been encoded in latent vectors, transconvolution layers were applied symmetrically to decode the latent vectors using the same hyperparameters used in the encoder. The size of the output was set to be equal to the size of the input data at the end of the decoder layer. Batch normalization and a hyperbolic tangent function were applied at the end of each layer. The clamped hyperbolic tangent was applied to the output of the decoder, which limited the output to values in the range of –1 to 1 and converted negative values to zeros. This caused the model to treat the distribution of negative values as a distribution of zeros. Finally, the recovered data, which were as close to the uncorrupted data as possible, were output.

Hyperparameters, filter size (m=20 and 30) and size of the latent vector (k=40, 60, and 80), were determined by a grid search. The other hyperparameters (the number of layers, q, and number of filters, n) were gradually increased and the best-performing values were selected. Stride was set adaptively according to the latent vector size.

For training and selecting the final hyperparameter settings, the training data set was used. The hyperparameters of the zero-inflated denoising convolutional autoencoder were tuned using 10-fold cross-validation. Hyperparameters exhibiting the lowest root mean square error (RMSE) were selected as the final hyperparameter settings.

When training the zero-inflated denoising convolutional autoencoder model, we used RMSE as a loss function to provide feedback to modify the weights. The loss between the original data and restored data was calculated for the entire record (imputed and original parts). Applying the loss function to all values allowed the zero-inflated denoising convolutional autoencoder to learn to impute the missing intervals and to reconstruct the other observed parts.

### Performance Evaluation

#### Metrics

Performance of the imputation methods were evaluated using two metrics on the imputed portion of the outputs. Partial RMSE


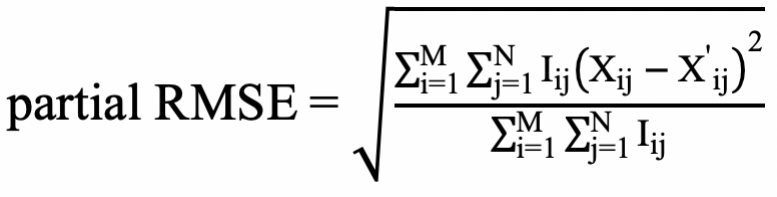


and partial MAE


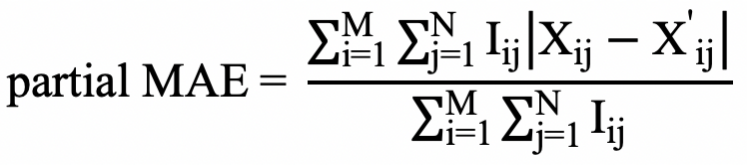


where


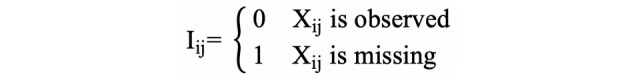


were calculated. In the equations, *i* and *j* represent the *i*th data record in a data set and the *j*th value in a data record, respectively. *I_ij_* was a binary variable that represented whether a variable was observed or missing, *M* was the number of records in the data set, *N* was the length of the data record, *X_ij_* indicated an original value, and *X'_ij_* indicated its imputed value. Lower values of partial RMSE and partial MAE indicated better imputation.

We calculated the standard deviation


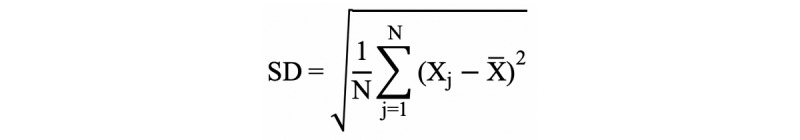


and the intradaily variability index


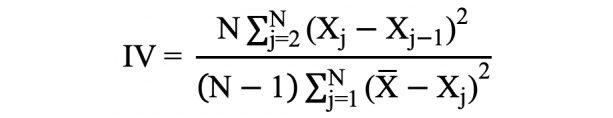


where *N* was the total number of values in the data record, *X_j_* was the *j*th value in the data record, and 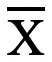 was the mean of the values in that record.

Intradaily variability index is a nonparametric index of the circadian rhythm and represents the fragmentation of activity. Index values range from 0 to 2, and higher values indicate higher variability.

To evaluate reconstruction of the original variability in the data, we calculated RMSE of the standard deviation,


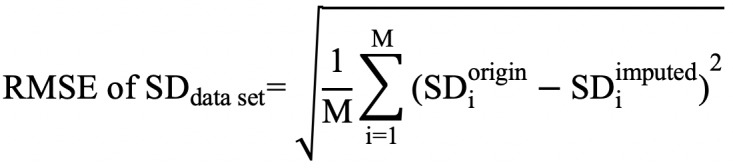


and RMSE of the intradaily variability,


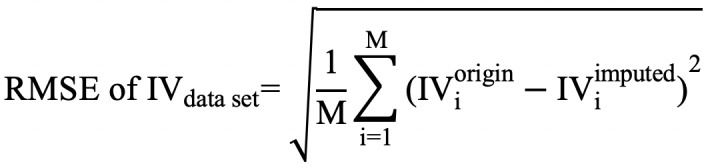


where *M* was the total number of records in a data set, *SD_i_^origin^* was the standard deviation of the *i*th original record, and *SD_i_^imputed^* was the standard deviation of the *i*th imputed record.

We also evaluated the RMSE in the missing intervals of a moderate-to-vigorous physical activity measure that was derived from the data. Moderate-to-vigorous physical was used to represent activity intensity. We applied a cutoff=1267 counts [35] to uniaxial data in NHANES and KNHANES and applied a cutoff=2691 counts [36], which was designed for a single magnitude vector, to the KCCDB data set. We measured the duration of moderate-to-vigorous physical activity using the 10-minute bout method, which identifies 10 minutes of moderate-to-vigorous physical activity if over 80% of the values are greater than the cutoff point [25]. We evaluated the moderate-to-vigorous physical activity measure from original and imputed data using the RMSE,


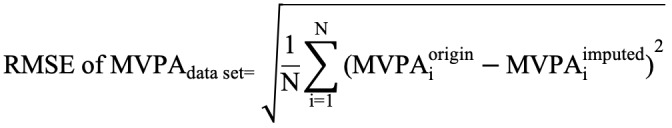


where, unlike the RMSE of intradaily variability or standard deviation, the duration of moderate-to-vigorous physical activity was calculated for only missing intervals. *M* is the total number of records in a data set, denotes the moderate-to-vigorous physical activity for the original values in the missing interval of *i*th data record, and *MVPA_i_^imputed^* denotes the moderate-to-vigorous physical activity for the imputed values in the missing interval of the *i*th data record*.*

#### Mean Imputation

The mean value at each minute was determined from the NHANES training set. For external validation, a model was constructed and evaluated for each data set without dividing them into training and testing sets.

#### Zero-Inflated Poisson Regression and Bayesian Regression Imputation

The multiple imputation-based models were constructed without dividing the data set into training, validation, and testing data sets. These models utilize the expectation–maximization algorithm, which requires the entire data set and fills in the missing values with values inferred from existing values in other data records. After imputation was performed, the performance was estimated using the NHANES test data set only. External validation was performed using the same process that was used to evaluate mean imputation.

#### Zero-Inflated Denoising Convolutional Autoencoder Imputation

The performance of zero-inflated denoising convolutional autoencoder was evaluated using the model generated by the training and validation sets of NHANES to impute the test set. For external validation, the performance was evaluated by applying the model trained on the NHANES data set to the external validation data sets without retraining.

## Results

To visualize the data and results, both Python (matplotlib, version 3.0.0; seaborn, version 0.9.0) and R (ggplot2) were used [37-39]. After preprocessing, the NHANES data set comprised 25,858 days of data from 9236 persons, the KNHANES data set comprised 3778 days of data from 1301 persons, and the KCCDB data set comprised 1829 days of data from 169 persons (Table 1).

After 10-fold cross-validation for hyperparameter tuning, we selected the hyperparameter conditions with the lowest mean RMSE (Table 2). Detailed results of 10-fold cross-validation are described in Multimedia Appendix 3.

We used the zero-inflated denoising convolutional autoencoder model with a filter length of 30 in the first convolutional layer and a latent vector size of 60 in the subsequent experiments in this study (q=10, n=8, m=30, k=60, Table 3).

**Table 1:** Baseline characteristics of the data sets.

Characteristics		NHANES^a^ (n=12,475)	KNHANES^b^ (n=1768)	KCCDB^c^ (n=177)
Age (years), mean (SD)		39.04 (22.27)	42.88 (13.04)	74.07 (7.05)
**Gender, n (%)**				
	Male	6077 (48.71)	662 (37.44)	56 (31.63)
	Female	6398 (51.28)	1106 (62.55)	121 (68.36)
Weight (kg), mean (SD)		75.26 (21.73)	63.35 (11.97)	59.03 (10.04)
Height (cm), mean (SD)		166.01 (11.72)	163.45 (8.55)	156.96 (8.33)
BMI (kg/m^2^), mean (SD)		27.03 (6.56)	23.62 (3.48)	22.66 (7.19)
Activity (count), mean (SD)		344 (694.23)	433 (586.78)	637 (1121.27)

^a^NHANES: National Health and Nutrition Examination Survey data set (device: ActiGraph AM-7164; type: uniaxial; sample rate: 0.016 Hz).

^b^KNHANES: Korea National Health and Nutrition Examination Survey data set (device: ActiGraph GTX3+; type: triaxial; sample rate: 0.016 Hz).

^c^KCCDB: Korean Chronic Cerebrovascular Disease Oriented Biobank data set (device: Fitmeter; type: triaxial; sample rate: 0.01 Hz).

**Table 2:** Result of 10-fold cross-validation.

	Hyperparameters		Result	
Experiment	Latent vector size, k	Filter size, m		RMSE^a^ (count), mean
1	40	20		830.5
2	40	30		838.0
3	60	20		858.7
4	60	30		788.4
5	80	20		825.1
6	80	30		831.0

^a^RMSE: root mean square error.

**Table 3:** Hyperparameter settings for the zero-inflated denoising convolutional autoencoder.

Component layer (order of layer)		Filter, n^a^ ×size (stride)	Feature map output size, n × size
Input			1×720
**Encoder**			
	Convolution (1)	8×30^b^ (2)	8×346
	Convolution (2)	16×20 (2)	16×164
	Convolution (3)	32×10 (2)	32×78
	Convolution (4)	64×10 (1)	64×69
	Convolution (5)	128×10 (1)	128×60^c^
**Decoder**			
	Transconvolution (6)	64×10 (1)	64×69
	Transconvolution (7)	32×10 (1)	32×78
	Transconvolution (8)	16×10 (2)	16×164
	Transconvolution (9)	8×20 (2)	8 ×346
	Transconvolution (10^d^)	1 ×30 (2)	1×720

^a^number of filters, n.

^b^filter size, m.

^c^latent vector size, k, extracted by the encoder.

^d^number of layers, q.

**Figure 4:**
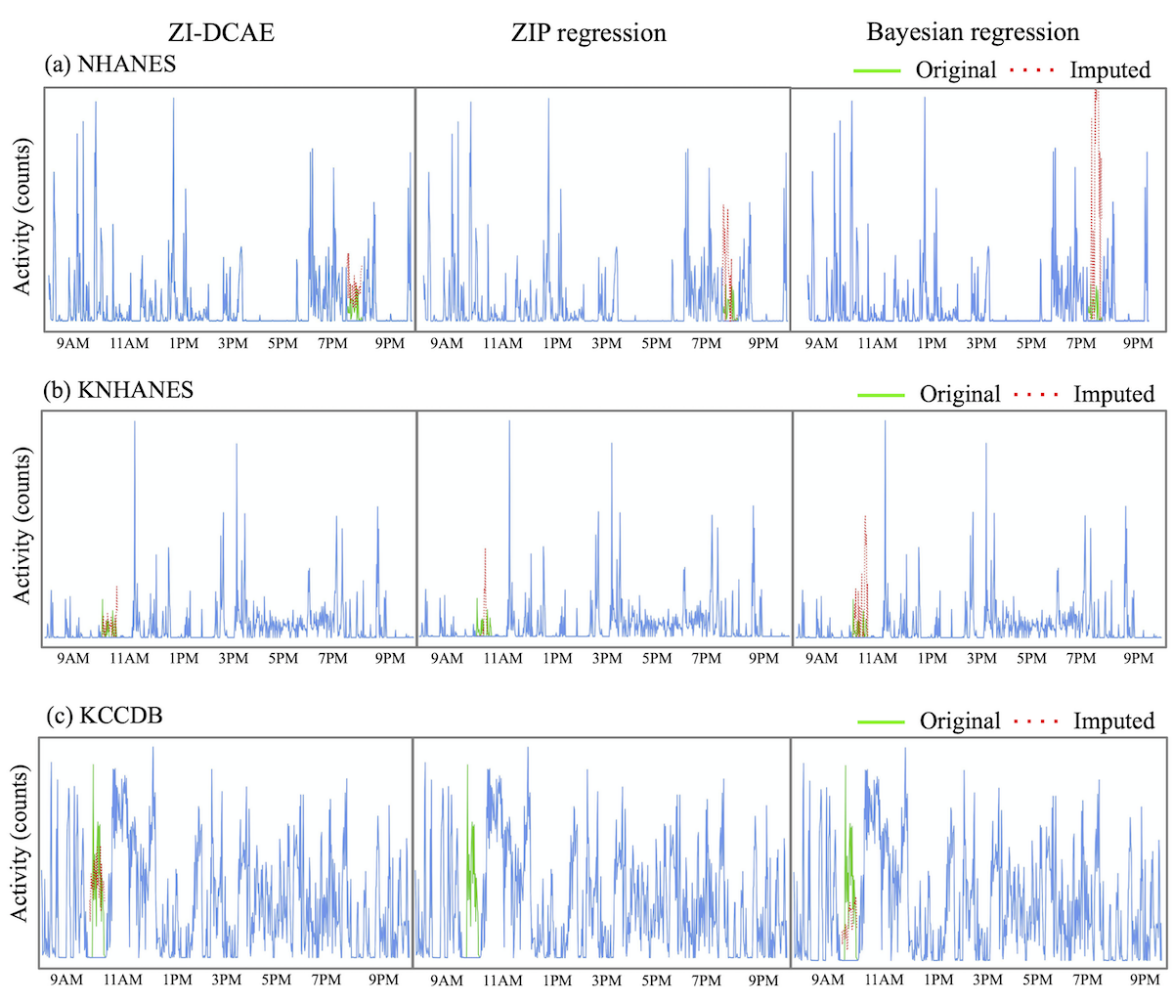
Examples of (a) NHANES, (b) KNHANES, and (c) KCCDB data sets for zero-inflated denoising convolutional autoencoder (left), zero-inflated Poisson regression (center), and Bayesian regression (right) with imputed (red) and original (green) intervals within the record (blue). KCCDB: Korean Chronic Cerebrovascular Disease Oriented Biobank; KNHANES: Korea National Health and Nutrition Examination Survey; ZI-DCAE: zero-inflated denoising convolutional autoencoder; ZIP: zero-inflated Poisson regression.

Examples for the zero-inflated denoising convolutional autoencoder, mean imputation, zero-inflated Poisson regression and Bayesian regression methods on the NHANES test data set are shown in Figure 4, and the results are given in Table 4. These results indicate that zero-inflated denoising convolutional autoencoder performed better and more accurately reflected the natural variation in human activity. In addition, the zero-inflated denoising convolutional autoencoder trained with the NHANES data set was tested on the KNHANES and KCCDB data set, and the results are also shown in Table 4. As with the results on the KNHANES and KCCDB data set, the values imputed by zero-inflated denoising convolutional autoencoder showed the lowest RMSE of standard deviation (24.4 counts and 27.1 counts, respectively).

In addition, we evaluated the RMSE of intradaily variability and moderate-to-vigorous physical activity for each data set to evaluate imputation. Zero-inflated denoising convolutional autoencoder yielded the lowest RMSE of intradaily variability for both the NHANES and KNHANES data sets with values of 0.047 and 0.037, respectively. In contrast, for KCCDB, zero-inflated denoising convolutional autoencoder had the second-lowest RMSE of intradaily variability with a value of 0.02. Moreover, the RMSE of moderate-to-vigorous physical activity for zero-inflated denoising convolutional autoencoder was the lowest (13.4 minutes) on KCCDB and the second lowest on the NHANES and KNHANES data sets (12.3 minutes and 12.9 minutes, respectively).

Additional analyses were performed including confidence interval calculation (Multimedia Appendix 4), additional experiments with 90- and 180-minute missing intervals (Multimedia Appendix 5 and Multimedia Appendix 6), other models (Multimedia Appendix 7), and other analyses (Multimedia Appendix 8 and Multimedia Appendix 9).

**Table 4:** Imputation performance results for the comparison methods.

Dataset measurement			ZI-DCAE^a^		Mean imputation		Zero-inflated Poisson regression		Bayesian regression
**NHANES^b^**									
	partial RMSE^c^ (counts)	839.3		1053.2		1255.6		924.5	
	partial MAE^d^ (counts)	431.1		545.4		508.5		605.8	
	RMSE of SD (counts)	35.1		65.2		69.2		34.2	
	RMSE of intradaily variability index	0.047		0.067		0.060		0.071	
	RMSE of moderate-to-vigorous physical activity (minutes)	12.3		16.2		16.2		11.0	
**KNHANES^e^**									
	partial RMSE (counts)	672.1		660.0		778.8		824.1	
	partial MAE (counts)	396.3		419.7		395.5		555.5	
	RMSE of SD (counts)	24.4		26.5		26.0		24.7	
	RMSE of intradaily variability index	0.037		0.039		0.040		0.050	
	RMSE of moderate-to-vigorous physical activity (minutes)	12.9		14.7		14.6		12.2	
**KCCDB^f^**									
	partial RMSE (counts)	1217.2		1313.2		1638.4		1139.4	
	partial MAE (counts)	819.6		1045.8		1161.6		810.7	
	RMSE of SD (counts)	27.1		30.8		29.6		27.7	
	RMSE of intradaily variability index	0.02		0.036		0.041		0.018	
	RMSE of moderate-to-vigorous physical activity (minutes)	13.4		14.9		14.9		13.6	

^a^ZI-DCAE: zero-inflated denoising convolutional autoencoder.

^b^NHANES: National Health and Nutrition Examination Survey data set.

^c^RMSE: root mean square error.

^d^MAE: mean absolute error.

^e^KNHANES: Korea National Health and Nutrition Examination Survey data set.

^f^KCCDB: Korean Chronic Cerebrovascular Disease Oriented Biobank data set.

## Discussion

### Principal Findings

Our study was the first to attempt to impute activity count data using a deep-learning approach. For activity data with intervals of zero values, in statistical models such as a zero-inflated Poisson distribution, the probability distribution of zeros is assumed to have a specific distribution. In contrast, the zero-inflated denoising convolutional autoencoder model created the distribution of zeros by using a clamped hyperbolic tangent activation function which caused the model to transform negative outputs to zero values. The zero-inflated denoising convolutional autoencoder model can learn the distribution of zeros from the data themselves. The results confirm that this approach performs better than previous approaches.

By testing the performance with an external data set that was not related to the training data set, we found that the model could be generalized. On the KNHANES and KCCDB data sets, our model exhibited better performance than those of the other imputation algorithms. Although our model was trained with the NHANES data set, which was collected with a uniaxial device, we confirmed that the zero-inflated denoising convolutional autoencoder model also worked well on triaxial data (the KCCDB data set). This result indicated that our model did not overfit to the NHANES data set, but instead was able to learn important features in the activity data.

In addition to predicting the missing values, our model was also able to reproduce the variability of activity data. On the NHANES data set, the zero-inflated denoising convolutional autoencoder had the second-lowest RMSE for standard deviation and the lowest RMSE for standard deviation on the external validation data sets. This characteristic is desirable for subsequent analysis using the imputed data set because some activity indices such as intradaily variability use variability in the activity data to evaluate the rhythms of human activity. We compared the RMSE of intradaily variability for the original data and imputed data. The zero-inflated denoising convolutional autoencoder showed a lower RMSE of intradaily variability than those of the other imputation methods. Generally, it seemed that the zero-inflated denoising convolutional autoencoder was able to restore the variability of the original data more accurately than other methods. These results suggest that the zero-inflated denoising convolutional autoencoder model can not only impute the missing data while reflecting the meaningful variability of the activity data in the general population (NHANES and KNHANES data sets), but can also reflect the variability of activity data from patients with cerebrovascular disease (KCCDB data set).

Moderate-to-vigorous physical activity is an index for evaluating the intensity of activity. We evaluated how well the model restored the duration of the original moderate-to-vigorous physical activity. With the lowest RMSE of moderate-to-vigorous physical activity in KCCDB and the second lowest in other data sets, the zero-inflated denoising convolutional autoencoder also demonstrated the ability to restore measures of activity intensity.

### Ad Hoc Fine-Tuning

Fine-tuning the approach could be considered to better reflect the unique characteristics of new data if there are enough complete cases available after training. To test whether fine-tuning could improve imputation performance, we conducted the following ad hoc analysis. The KNHANES data set was split into training, validation, and testing data sets in a 9:1:1 ratio. The fine-tuning process was conducted with the training data of the KNHANES data set using the fully developed model used in this study. Training was stopped when the performance of the model was best in the KNHANES validation set. When the performance was evaluated on the KNHANES testing set, the fine-tuned model performed better, with a partial RMSE of 663.6 counts, partial MAE of 391.2 counts, whereas those of the baseline model were 672.1 counts, 396.3 counts, respectively.

### Limitations

There are some limitations to be discussed. First, activity patterns can depend on demographic factors such as age, BMI, and other metrics. Although demographic information may help improve the performance of the imputation model, it was not used in this study because of a lack of data. If we can obtain sufficient data sets with demographic information in future, we will be able to improve the performance of our imputation model by including it in the training; however, because the demographic information of the user is often unknown in actual studies, it could be more practical for the model to restore missing data using only activity data. Second, some activities that cause participants to remove the device cannot be collected in data set, and these data are difficult to impute correctly. This limitation is always carefully considered before imputation methods are applied. Although all imputation methods have this limitation, our model performed better than other methods did. Third, although many imputation methods exist, we only compared our model with three methods. We conducted Gaussian process regression, but it predicted only zero values for the imputed data (Multimedia Appendix 9). Further studies should include a wider range of imputation methods. Finally, the use of a count-based algorithm to define missing intervals could be too simple for representing nonwear time; however, the aim of our work to impute missing intervals, and we only utilized a count-based nonwear detection algorithm to construct complete data sets. Regardless of which nonwear detection algorithms is used to generate corruption, the model should be able learn the pattern and impute the corrupted data.

### Conclusions

To our knowledge, this is the first study to develop a deep-learning model for imputing missing values in actigraphy data. The results of this study suggest that the deep learning approach is useful for imputing missing values in activity data. We expect that our model will contribute to studies of human activity by decreasing the amount of discarded data due to missing values.

## Appendix

Multimedia Appendix 1 Detailed information about accelerometer device.

Multimedia Appendix 2 The distribution of length of consecutive zeros in each dataset.

Multimedia Appendix 3 Detailed result of 10-fold cross validation.

Multimedia Appendix 4 Confidence intervals of evaluation measurement by the bootstrapping method.

Multimedia Appendix 5 Experimental results for 90- and 180-min missing intervals for each imputation method.

Multimedia Appendix 6 Example imputed result with 90- and 180-min missing intervals.

Multimedia Appendix 7 Comparison of model performance between ZI-DCAE model and the naïve convolutional autoencoder with sigmoid function.

Multimedia Appendix 8 Accuracy of restoring Moderate-to-Vigorous Physical Activity (MVPA).

Multimedia Appendix 9 The result of Gaussian process imputation.

## Abbreviations

BMI: body mass index
KCCDB: Korean Chronic Cerebrovascular Disease Oriented Biobank
KNHANES: Korea National Health and Nutrition Examination Survey
MAE: mean absolute error
NHANES: National Health and Nutrition Examination Survey
RMSE: root mean square error

## References

[1] Tranah GJ, Blackwell T, Stone KL, Ancoli-Israel S, Paudel ML, Ensrud KE, Cauley JA, Redline S, Hillier TA, Cummings SR, Yaffe K, Research Group SOF (2011). Circadian activity rhythms and risk of incident dementia and mild cognitive impairment in older women. Ann Neurol.

[2] Kinder JR, Lee KA, Thompson H, Hicks K, Topp K, Madsen KA (2012). Validation of a hip-worn accelerometer in measuring sleep time in children. J Pediatr Nurs.

[3] Ravi N, Dandekar N, Mysore P, Littman M Activity recognition from accelerometer data.

[4] Shephard RJ (2003). Limits to the measurement of habitual physical activity by questionnaires. Br J Sports Med.

[5] Zhu W, Wadley VG, Howard VJ, Hutto B, Blair SN, Hooker SP (2017). Objectively measured physical activity and cognitive function in older adults. Med Sci Sports Exerc.

[6] Cho C, Lee T, Kim M, In HP, Kim L, Lee H (2019). Mood prediction of patients with mood disorders by machine learning using passive digital phenotypes based on the circadian rhythm: prospective observational cohort study. J Med Internet Res.

[7] Witting W, Kwa IH, Eikelenboom P, Mirmiran M, Swaab DF (1990). Alterations in the circadian rest-activity rhythm in aging and Alzheimer's disease. Biol Psychiatry.

[8] Catellier DJ, Hannan PJ, Murray DM, Addy CL, Conway TL, Yang S, Rice JC (2005). Imputation of missing data when measuring physical activity by accelerometry. Med Sci Sports Exerc.

[9] Yue Xu S, Nelson S, Kerr J, Godbole S, Patterson R, Merchant G, Abramson I, Staudenmayer J, Natarajan L (2018). Statistical approaches to account for missing values in accelerometer data: applications to modeling physical activity. Stat Methods Med Res.

[10] Cheng Y, Wang F, Zhang P, Hu J (2016). Risk prediction with electronic health records: a deep learning approach. https://epubs.siam.org/doi/10.1137/1.9781611974348.49.

[11] Gulshan V, Peng L, Coram M, Stumpe MC, Wu D, Narayanaswamy A, Venugopalan S, Widner K, Madams T, Cuadros J, Kim R, Raman R, Nelson PC, Mega JL, Webster DR (2016). Development and validation of a deep learning algorithm for detection of diabetic retinopathy in retinal fundus photographs. JAMA.

[12] Yoon D, Lim HS, Jung K, Kim TY, Lee S (2019). Deep learning-based electrocardiogram signal noise detection and screening model. Healthc Inform Res.

[13] Baldiditor (2012). Autoencoders, unsupervised learning, and deep architectures. Proceedings of ICML workshop on unsupervised and transfer learning.

[14] Duan Y, Lv Y, Liu Y, Wang F (2016). An efficient realization of deep learning for traffic data imputation. Transportation Research Part C: Emerging Technologies.

[15] Wang L (2016). Recognition of human activities using continuous autoencoders with wearable sensors. Sensors (Basel).

[16] National Center for Health Statistics (2003). Centers for Disease Control and Prevention. National Health and Nutrition Examination Survey Data.

[17] National Center for Health Statistics (2005). Centers for Disease Control and Prevention. National Health and Nutrition Examination Survey Data.

[18] Kweon Sanghui, Kim Yuna, Jang Myoung-jin, Kim Yoonjung, Kim Kirang, Choi Sunhye, Chun Chaemin, Khang Young-Ho, Oh Kyungwon (2014). Data resource profile: the Korea National Health and Nutrition Examination Survey (KNHANES). Int J Epidemiol.

[19] (2014). The Sixth Korea National Health and Nutrition Examination Survey (KNHANES VI-2). Korea Centers for Disease Control and Prevention.

[20] (2015). The Sixth Korea National Health and Nutrition Examination Survey (KNHANES VI-3). Korea Centers for Disease Control and Prevention.

[21] Survey Overview of Korean National Health and Nutrition Examination (KNHANES). Korea Centers for Disease Control and Prevention.

[22] Kozey SL, Staudenmayer JW, Troiano RP, Freedson PS (2010). Comparison of the ActiGraph 7164 and the ActiGraph GT1M during self-paced locomotion. Med Sci Sports Exerc.

[23] Cradock AL, Wiecha JL, Peterson KE, Sobol AM, Colditz GA, Gortmaker SL (2004). Youth recall and TriTrac accelerometer estimates of physical activity levels. Med Sci Sports Exerc.

[24] Evenson KR (2011). Towards an understanding of change in physical activity from pregnancy through postpartum. Psychol Sport Exerc.

[25] Metzger JS, Catellier DJ, Evenson KR, Treuth MS, Rosamond WD, Siega-Riz AM (2008). Patterns of objectively measured physical activity in the United States. Med Sci Sports Exerc.

[26] Pratama I, Permanasari A, Ardiyanto I, Indrayani R (2016). A review of missing values handling methods on time-series data.

[27] Azur MJ, Stuart EA, Frangakis C, Leaf PJ (2011). Multiple imputation by chained equations: what is it and how does it work?. Int J Methods Psychiatr Res.

[28] Lee JA, Gill J (2018). Missing value imputation for physical activity data measured by accelerometer. Stat Methods Med Res.

[29] Jung AL( accelmissing: Missing Value Imputation for Accelerometer Data. R package version 1.

[30] Vincent P, Larochelle H, Bengio Y, Manzagol P, editors (2008). Extractingcomposing robust features with denoising autoencoders.

[31] Krizhevsky A, Sutskever I, Hinton GE (2017). ImageNet classification with deep convolutional neural networks. Commun. ACM.

[32] Abdel-Hamid O, Mohamed A, Jiang H, Deng L, Penn G, Yu D (2014). Convolutional neural networks for speech recognition. IEEE/ACM Trans. Audio Speech Lang. Process.

[33] Kiranyaz S, Ince T, Gabbouj M (2016). Real-time patient-specific ECG classification by 1-D convolutional neural networks. IEEE Trans Biomed Eng.

[34] PyTorch (version 1.4.1).

[35] Leenders NYJM, Nelson TE, Sherman WM (2003). Ability of different physical activity monitors to detect movement during treadmill walking. Int J Sports Med.

[36] Freedson PS, Melanson E, Sirard J (1998). Calibration of the Computer Science and Applications, Inc. accelerometer. Med Sci Sports Exerc.

[37] Visualization with Python. matplotlib.

[38] Statistical data visualization. seaborn.

[39] ggplot2.

