# Supplementary material for: Deep Learning Approach for Imputation of Missing Values in Actigraphy Data: Algorithm Development Study
Source: JMIR Mhealth Uhealth. 2020 Jul 23;8(7):e16113. doi: 10.2196/16113 (PMC7413283; doi:10.2196/16113)
Supplement: Multimedia Appendix 1 [file mhealth_v8i7e16113_app1.docx]

# **Multimedia Appendix 1.** Detailed information about the accelerometer devices

- NHANES dataset


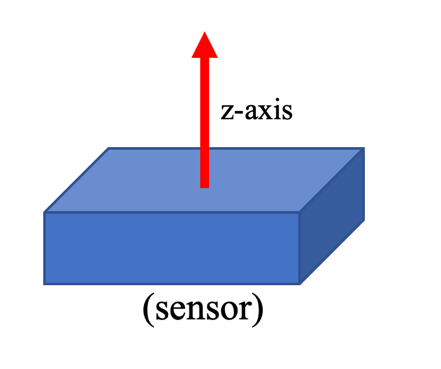


**Figure S1-1**. Example of uni-axial accelerometer device

The NHANES accelerometer dataset was collected using an uni-axial accelerometer device (ActiGraph AM-7164 device). As shown in the figure above, in a uni-axial accelerometer device, the acceleration values are collected from the vertical axis (z-axis) of the instrument

- KNHANES, KCCDB dataset


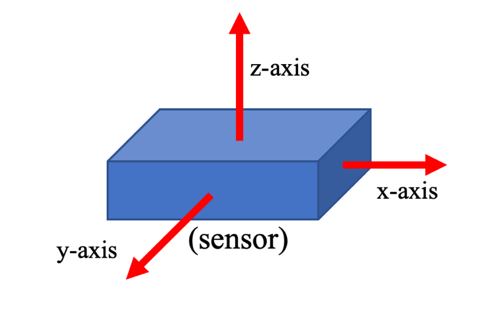


**Figure S1-2**. Example of tri-axial accelerometer device

The KNHANES and KCCDB accelerometer datasets were collected by tri-axial accelerometer devices (KNHANES: Actigraph GT3X+, KCCDB: Fit.Life Fitmeter). Unlike the uni-axial accelerometer device describe above, a tri-axial accelerometer device collects acceleration values from the x-, y-, and z-axes. However, in the KNHANES accelerometer dataset, only vertical axis data (z-axis data) has been made publicly available.

In this paper, data from each device are used in their original format and values from one device are not comparable to those from others since activity units, device type, and device range (± g) vary.
