# Supplementary material for: Deep Learning Approach for Imputation of Missing Values in Actigraphy Data: Algorithm Development Study
Source: JMIR Mhealth Uhealth. 2020 Jul 23;8(7):e16113. doi: 10.2196/16113 (PMC7413283; doi:10.2196/16113)
Supplement: Multimedia Appendix 2 [file mhealth_v8i7e16113_app2.docx]

# **Multimedia Appendix 2.** Distribution of the lengths of consecutive zeros in each dataset

**Table S2.** Portion of data containing missing intervals according to the definition of 30 minutes of consecutive zeros

| Dataset | # of total data per day | # of data containing non-wear time  (non-wear over 30 minutes) | % of incomplete data |
| --- | --- | --- | --- |
| NHANES | 101,514 | 75,656 | 74.52 |
| KNHANES | 12,376 | 8,598 | 69.47 |
| KCCDB | 4,779 | 2,951 | 61.75 |

In Table S2, we compare portion of data with missing intervals in each dataset. First, we note that the NHANES dataset has a larger number of data containing non-wear time (75,656) and a higher ratio of incomplete data (74.62%) than the KNHANES dataset (8,598 and 69.47%, respectively) and the KCCDB dataset (2,951 and 61.75%, respectively).

**Figure S2**. Distribution of lengths of consecutive zeros in the KNHANES and KCCDB datasets

In Figure S2, we present the distribution of consecutive zeros in the KNHANES and KCCDB datasets. In each histogram, the x-axis shows the length of consecutive zeros and the y-axis is the number of zero intervals. This figure confirms that the frequency of missing intervals less than 30 minutes was the highest in both datasets
