# Supplementary material for: Deep Learning Approach for Imputation of Missing Values in Actigraphy Data: Algorithm Development Study
Source: JMIR Mhealth Uhealth. 2020 Jul 23;8(7):e16113. doi: 10.2196/16113 (PMC7413283; doi:10.2196/16113)
Supplement: Multimedia Appendix 3 [file mhealth_v8i7e16113_app3.docx]

# **Multimedia Appendix 3.** Detailed result of 10-fold cross validation

**Table S3.** Detailed result of 10-fold cross validation

|  | | Condition | | | | | |
| --- | --- | --- | --- | --- | --- | --- | --- |
|  | |  |  |  |  |  |  |
| **Latent size** | |  |  |  |  |  |  |
|  | | 40 | 40 | 60 | 60 | 80 | 80 |
| **Filter size** | |  |  |  |  |  |  |
|  | | 20 | 30 | 20 | 30 | 20 | 30 |
| **Fold** | | RMSE | | | | | |
|  | **1** |  |  |  |  |  |  |
|  |  | 916 | 931 | 926 | 906 | 908 | 910 |
|  | **2** |  |  |  |  |  |  |
|  |  | 885 | 886 | 910 | 868 | 881 | 872 |
|  | **3** |  |  |  |  |  |  |
|  |  | 832 | 835 | 842 | 806 | 821 | 822 |
|  | **4** |  |  |  |  |  |  |
|  |  | 829 | 832 | 863 | 791 | 822 | 826 |
|  | **5** |  |  |  |  |  |  |
|  |  | 798 | 802 | 825 | 770 | 795 | 806 |
|  | **6** |  |  |  |  |  |  |
|  |  | 808 | 816 | 838 | 768 | 806 | 809 |
|  | **7** |  |  |  |  |  |  |
|  |  | 770 | 781 | 804 | 709 | 761 | 775 |
|  | **8** |  |  |  |  |  |  |
|  |  | 806 | 820 | 842 | 756 | 809 | 813 |
|  | **9** |  |  |  |  |  |  |
|  |  | 836 | 847 | 882 | 769 | 830 | 846 |
|  | **10** |  |  |  |  |  |  |
|  |  | 825 | 830 | 855 | 741 | 818 | 831 |
| **Mean** | |  |  |  |  |  |  |
|  | | 830.5 | 838 | 858.7 | 788.4 | 825.1 | 831 |
| **StdDev** | |  |  |  |  |  |  |
|  | | 42.38 | 42.81 | 37.78 | 58.89 | 41.81 | 37.74 |
