# Supplementary material for: Deep Learning Approach for Imputation of Missing Values in Actigraphy Data: Algorithm Development Study
Source: JMIR Mhealth Uhealth. 2020 Jul 23;8(7):e16113. doi: 10.2196/16113 (PMC7413283; doi:10.2196/16113)
Supplement: Multimedia Appendix 4 [file mhealth_v8i7e16113_app4.docx]

# **Multimedia Appendix 4.** Confidence intervals of evaluation measurement by the bootstrapping method. To confirm whether the results of the evaluation are valid, we calculated 95% confidence intervals for each of the three models except mean imputation methods. The confidence intervals were calculated by bootstrapping methods, where 70% of the data were sampled in each data set for every bootstrap.

**Figure S4. 95% CIs of the evaluation measurement using the bootstrapping method**

PRMSE, partial root mean squared error; PMAE, partial mean absolute error; RMSE of SD, root mean squared error of the standard deviation: RMSE of IV, root mean squared error of the intra-daily variability;

We calculated the CIs to confirm that the differences in performance obtained by the experiments are valid. Bootstrapping methods were used to calculate the CIs. Because this calculation was repeated 50 times and resampled 70% of training data before evaluating the performance, the results are worse than the results of Table 4 in the main paper overall. However, the tendency of the CIs follow those of the evaluation of the models in the paper, and we believe that this indicates that ZI-DCAE validly enhances performance.
