# Supplementary material for: Deep Learning Approach for Imputation of Missing Values in Actigraphy Data: Algorithm Development Study
Source: JMIR Mhealth Uhealth. 2020 Jul 23;8(7):e16113. doi: 10.2196/16113 (PMC7413283; doi:10.2196/16113)
Supplement: Multimedia Appendix 5 [file mhealth_v8i7e16113_app5.docx]

# **Multimedia Appendix 5.** Experimental results for 90- and 180-min missing intervals for each imputation method

Longer missing intervals led to lower model performances; however, we found that the zero-inflated denoising convolutional autoencoder still performed better. With 90-minute missing interval, zero-inflated denoising convolutional autoencoder produced the lowest partial RMSE, RMSE of standard deviation, and RMSE of intradaily variability on NHANES. On KNHANES, zero-inflated denoising convolutional autoencoder shows the best performance in partial RMSE and RMSE of intradaily variability. Finally, we noted that the partial RMSE, partial MAE, and RMSE of intradaily variability obtained by zero-inflated denoising convolutional autoencoder were lower than those obtained by other methods. The results for 180-minute missing intervals had similar patterns to those of the 90-minute missing interval experiments.

**Table S5-1.** Imputation performance results for the methods for 90-min missing intervals

| Datasets | Measurement | ZI-DCAE | Mean imputation | ZIP regression | Bayesian regression |
| --- | --- | --- | --- | --- | --- |
|  |  |  |  |  |  |
| NHANES^a^ |  |  |  |  |  |
|  | PRMSE (cpm) | 878.6 | 1093.7 | 1221.4 | 1109.6 |
|  | PMAE (cpm) | 497.3 | 549.2 | 484.7 | 668.2 |
|  | RMSE of SD^b^ (cpm) | 90.2 | 147.5 | 169.0 | 108.0 |
|  | RMSE of IV^c^ | 0.08 | 0.13 | 0.096 | 0.121 |
|  | RMSE of MVPA^d^ (min) | 22.5 | 29.0 | 29.1 | 21.4 |
| KNHANES |  |  |  |  |  |
|  | PRMSE (cpm) | 708.0 | 708.0 | 811.1 | 895.8 |
|  | PMAE (cpm) | 430.8 | 431.7 | 383.5 | 608.7 |
|  | RMSE of SD^b^ (cpm) | 75.1 | 76.9 | 72.4 | 69.9 |
|  | RMSE of IV^c^ | 0.078 | 0.086 | 0.087 | 0.108 |
|  | RMSE of MVPA^d^ (min) | 23.1 | 24.2 | 24.1 | 22.93 |
| KCCDB |  |  |  |  |  |
|  | PRMSE (cpm) | 1270.2 | 1303.4 | 1761.6 | 1476.7 |
|  | PMAE (cpm) | 942.6 | 1039.0 | 1247.4 | 1002.8 |
|  | RMSE of SD^b^ (cpm) | 88.5 | 89.7 | 76.3 | 73.8 |
|  | RMSE of IV^c^ | 0.042 | 0.077 | 0.092 | 0.054 |
|  | RMSE of MVPA^d^ (min) | 24.9 | 23.6 | 23.6 | 23.6 |

^a^Test dataset of NHANES

^b^Root mean square error of standard deviation for a dataset

^c^Root mean square error of intra-daily variability for a dataset

^d^Root mean square error of Moderate-to-Vigorous Physical Activity for a dataset

PRMSE, partial root mean squared error; PMAE, partial mean absolute error;

We found that ZI-DCAE obtained the best results for the PRMSE, PMAE, RMSE of SD and RMSE of IV on NHANES. On the validation dataset, ZI-DCAE has the lowest PRMSE and RMSE of IV, but still performs better overall. The results for 180-min imputation intervals show a similar pattern with those of the 90-min imputation interval experiment.

On NHANES, the PRMSE of ZI-DCAE (263.0 cpm) was lower than that of other methods on average. Moreover, its RMSE of SD (51.3 cpm) and RMSE of IV (0.035) were lower than those of other methods. With respect to PMAE, ZIP regression obtained the lowest value (484.7 cpm) and that of ZI-DCAE was 497.3 cpm. With the exception of PMAE, the ZI-DCAE model obtained better performance scores than did the other methods.

On KNHANES, on average, the PRMSE of ZI-DCAE (96.6 cpm) was lower than those of other methods. Moreover, its RMSE of IV (0.015) was lower than those of other methods. ZI-DCAE performs better than other models with respect to these metrics. In contrast, for PMAE and the RMSE of SD, ZIP regression and Bayesian regression obtained better performance, respectively.

On KCCDB, on average, the PRMSE and PMAE of ZI-DCAE were lower (243.3 cpm and 154.0 cpm, respectively) than those of other methods. Moreover, on average, the RMSE of IV obtained by ZI-DCAE (0.032) was lower than those of other methods. In contrast, with respect to the RMSE of SD, Bayesian regression obtained the lowest value (73.8 cpm). Those of ZI-DCAE, mean imputation, and ZIP regression were 88.5 cpm, 89.7 cpm, and 76.3 cpm, respectively.

Consequently, although ZI-DCAE obtains lower values for a few evaluation indicators, the ZI-DCAE model obtained better imputation performance than other methods for most results.

**Table S5-2.** Imputation performance results for the methods with 180-min missing intervals

| Datasets | Measurement | ZI-DCAE | Mean imputation | ZIP regression | Bayesian regression |
| --- | --- | --- | --- | --- | --- |
|  |  |  |  |  |  |
| NHANES^a^ |  |  |  |  |  |
|  | PRMSE (cpm) | 887.2 | 1063.9 | 1180.2 | 1120.3 |
|  | PMAE (cpm) | 515.1 | 551.1 | 477.6 | 682.9 |
|  | RMSE of SD^b^ (cpm) | 164.4 | 238.2 | 245.0 | 184.5 |
|  | RMSE of IV^c^ | 0.124 | 0.196 | 0.147 | 0.167 |
|  | RMSE of MVPA^d^ (min) | 36.8 | 42.0 | 42.0 | 29.7 |
| KNHANES |  |  |  |  |  |
|  | PRMSE (cpm) | 692.2 | 711.8 | 783.1 | 899.0 |
|  | PMAE (cpm) | 437.5 | 426.6 | 372.8 | 621.3 |
|  | RMSE of SD^b^ (cpm) | 119.4 | 121.2 | 108.7 | 100.1 |
|  | RMSE of IV^c^ | 0.115 | 0.124 | 0.129 | 0.147 |
|  | RMSE of MVPA^d^ (min) | 28.8 | 28.2 | 28.1 | 26.8 |
| KCCDB |  |  |  |  |  |
|  | PRMSE (cpm) | 1290.7 | 1301.5 | 1765.8 | 1523.4 |
|  | PMAE (cpm) | 940.9 | 1040.3 | 1228.4 | 1037.5 |
|  | RMSE of SD^b^ (cpm) | 165.7 | 168.2 | 122.2 | 131.4 |
|  | RMSE of IV^c^ | 0.072 | 0.116 | 0.150 | 0.170 |
|  | RMSE of MVPA^d^ (min) | 33.1 | 34.2 | 34.1 | 34.1 |

^a^Test dataset of NHANES

^b^Root mean square error of standard deviation for a dataset

^c^Root mean square error of intra-daily variability for a dataset

^d^Root mean square error of Moderate-to-Vigorous Physical Activity for a dataset

PRMSE, partial root mean squared error; PMAE, partial mean absolute error;

On the NHANES dataset, on average, the PRMSE of ZI-DCAE was 234.0 cpm lower than those of other methods. For the RMSE of SD and RMSE of IV, they were lower (58.4 cpm and 0.046, respectively) than those of other methods. The PMAE of SD obtained by ZIP regression was the lowest (477.6 cpm).

On the KNHANES dataset, the PRMSE of ZI-DCAE was 105.9 cpm, which was lower than that of other methods on average. The RMSE of IV obtained by ZI-DCAE was 0.115, which is the lowest value. With respect to the PMAE and RMSE of SD, ZIP regression obtained the lowest PMAE value (372.8 cpm) and Bayesian regression obtained the lowest RMSE of SD value (100.1 cpm).

On the KDDCB dataset, on average, the PRMSE and PMAE of ZI-DCAE were 239.6 cpm and 161.6 cpm, respectively. Moreover, the RMSE of IV from ZI-DCAE was 0.073 cpm, which was lower than those of other methods. For RMSE of SD, ZIP regression obtained the lowest value (122.2 cpm). Otherwise, ZI-DCAE obtained the lowest values for all other metrics.
