# Supplementary material for: Deep Learning Approach for Imputation of Missing Values in Actigraphy Data: Algorithm Development Study
Source: JMIR Mhealth Uhealth. 2020 Jul 23;8(7):e16113. doi: 10.2196/16113 (PMC7413283; doi:10.2196/16113)
Supplement: Multimedia Appendix 6 [file mhealth_v8i7e16113_app6.docx]

# **Multimedia Appendix 6.** Example imputed result with 90- and 180-min missing intervals

In Figures S6-1 and S6-2, we visualize the data imputed from each imputation method for 90-min and 180-min missing intervals. In each figure, the x-axis is the collection time of the accelerometer data and the y-axis is the value of the accelerometer (cpm). The blue line indicates the original accelerometer data without imputation and the green line indicates the part of the original data that requires imputation. The red line indicates the imputed data.


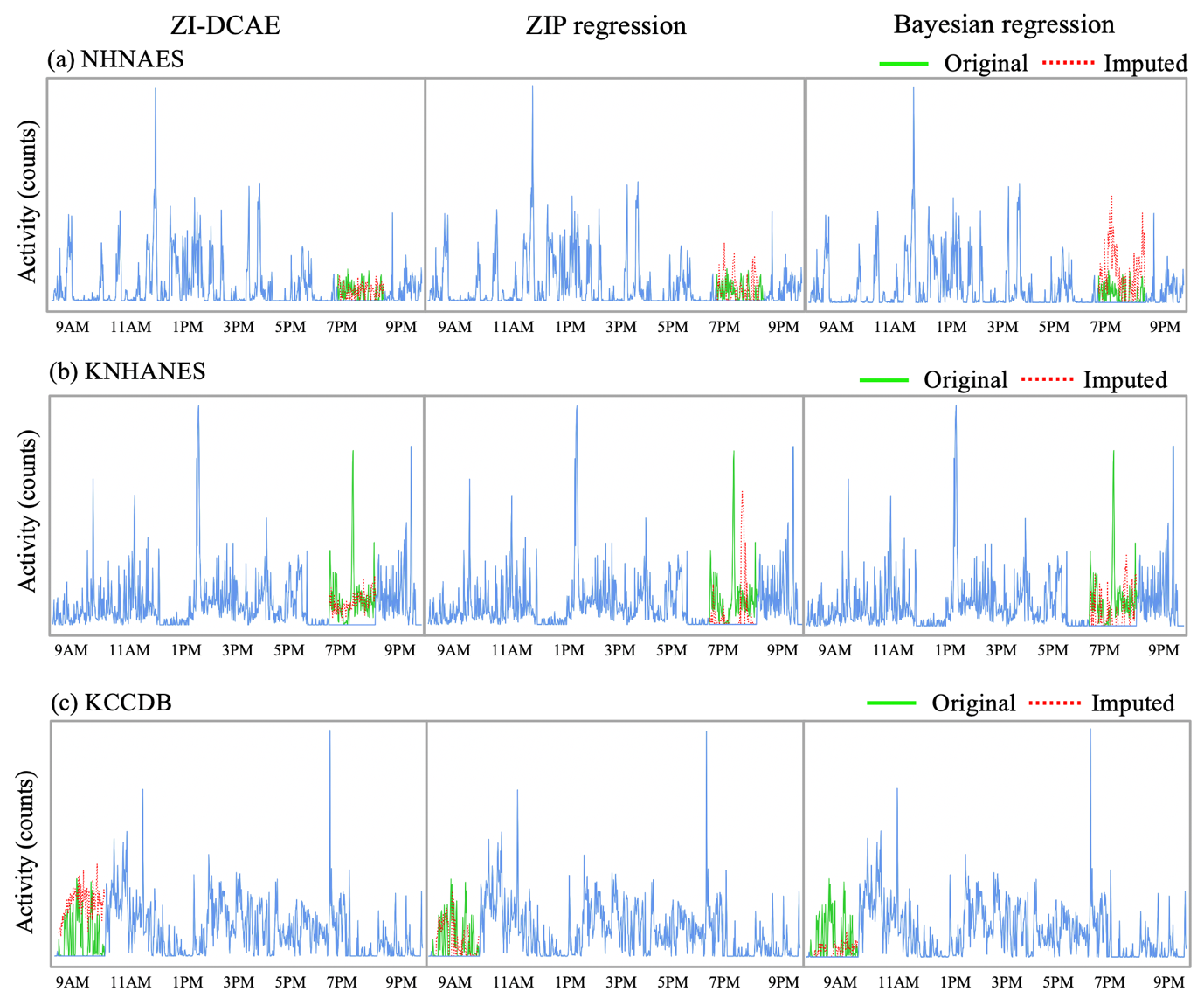


**Figure S6-1.** Example of imputation results for the ZI-DCAE, ZIP regression, and Bayesian regression methods for 90-min missing intervals


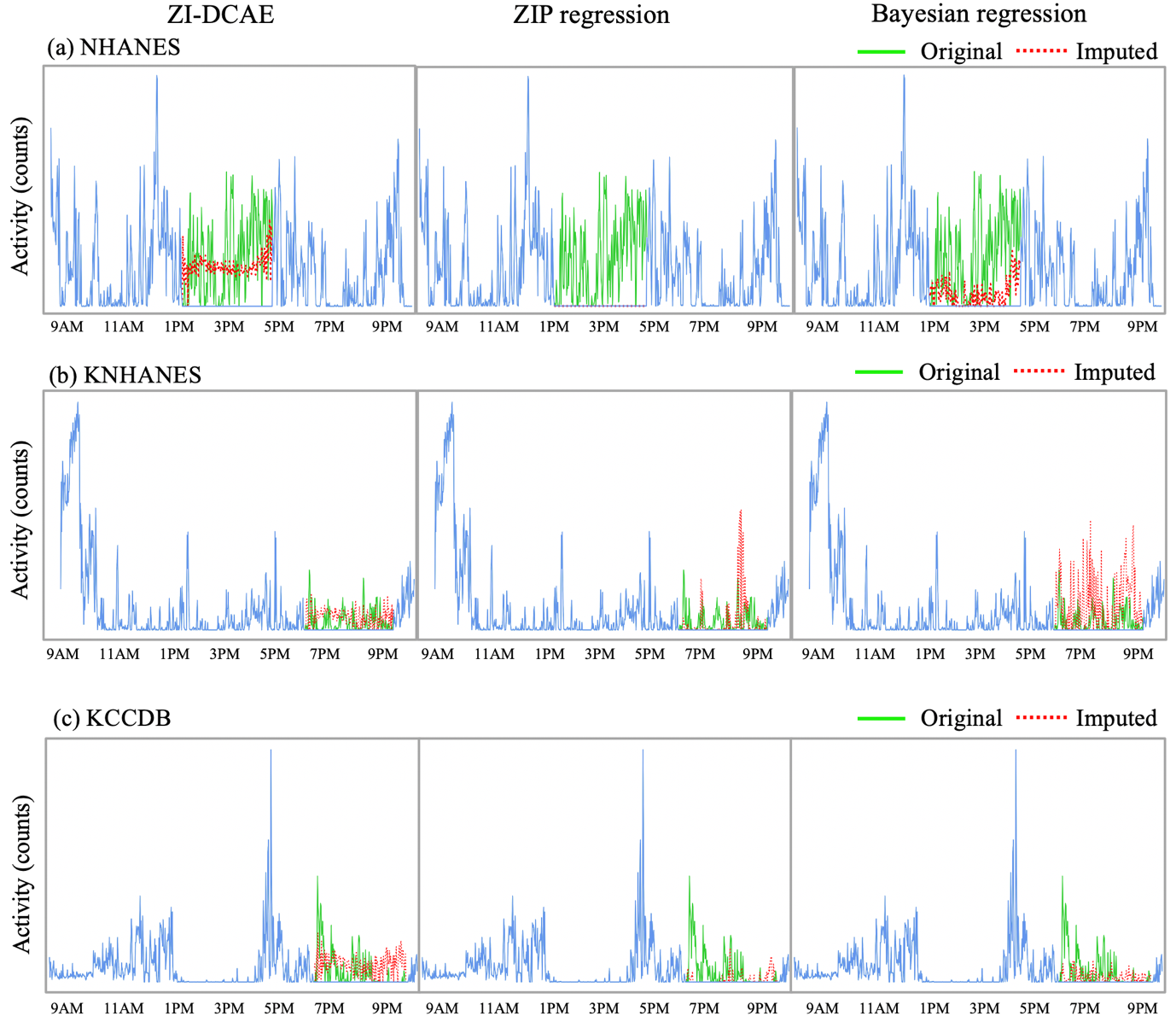


**Figure S6-2.** Example of imputation results for the ZI-DCAE, ZIP regression, and Bayesian regression methods for 180-min missing intervals
