# Supplementary material for: Deep Learning Approach for Imputation of Missing Values in Actigraphy Data: Algorithm Development Study
Source: JMIR Mhealth Uhealth. 2020 Jul 23;8(7):e16113. doi: 10.2196/16113 (PMC7413283; doi:10.2196/16113)
Supplement: Multimedia Appendix 7 [file mhealth_v8i7e16113_app7.docx]

# **Multimedia Appendix 7.** Comparison of model performance between ZI-DCAE model and the naïve convolutional autoencoder with sigmoid function

**Table S7-1.** Comparison of ZI-DCAE model performance with respect to a naïve convolutional autoencoder with a sigmoid function

| Datasets | Measurement | 30 minutes  missing interval | | 90 minutes  missing interval | | 180 minutes  missing interval | |
| --- | --- | --- | --- | --- | --- | --- | --- |
|  |  | Naïve sigmoid^a^ | ZI-DCAE | Naïve sigmoid^a^ | ZI-DCAE | Naïve sigmoid^a^ | ZI-DCAE |
| NHANES^b^ |  |  | |  | |  | |
|  | PRMSE | 886.2 | 839.3 | 887.5 | 878.6 | 896.6 | 887.2 |
|  | PMAE | 536.2 | 431.1 | 523.7 | 497.3 | 518.5 | 515.1 |
|  | RMSE of SD^c^ | 37.3 | 35.1 | 92.1 | 90.2 | 166.9 | 164.4 |
|  | RMSE of IV^d^ | 0.046 | 0.047 | 0.081 | 0.08 | 0.123 | 0.124 |
| KNHANES |  |  |  |  |  |  |  |
|  | PRMSE | 678.2 | 672.1 | 711.5 | 708.0 | 689.0 | 692.2 |
|  | PMAE | 446.7 | 396.3 | 449.2 | 430.8 | 436.1 | 437.5 |
|  | RMSE of SD^c^ | 25.0 | 24.4 | 75.5 | 75.1 | 120.1 | 119.4 |
|  | RMSE of IV^d^ | 0.035 | 0.037 | 0.078 | 0.078 | 0.112 | 0.115 |
| KCCDB |  |  |  |  |  |  |  |
|  | PRMSE | 1,251 | 1,217.2 | 1,293.2 | 1,270.2 | 1,301.0 | 1,290.7 |
|  | PMAE | 949.2 | 819.6 | 960.2 | 942.6 | 940.4 | 940.9 |
|  | RMSE of SD^c^ | 27.7 | 27.1 | 87.5 | 88.5 | 163.3 | 167.5 |
|  | RMSE of IV^d^ | 0.019 | 0.02 | 0.043 | 0.042 | 0.073 | 0.072 |

^a^Naïve convolutional autoencoder with sigmoid

^b^Test dataset of NHANES

^c^Root mean square error of standard deviation for a dataset

^d^Root mean square error of intra-daily variability for a dataset

PRMSE, partial root mean squared error; PMAE, partial mean absolute error;

We compared the performance of ZI-DCAE with that of a naïve convolutional autoencoder with a sigmoid activation function. Because we applied min-max normalization, the reconstructed output ranges from zero to one. A sigmoid activation function is usually utilized to limit the output range to between zero and one.

As a result, on average, the PRMSE and PRMAE of ZI-DCAE are 18.50 cpm and 14.25 cpm, which are lower than those of the naïve convolutional autoencoder with the sigmoid activation function. Although the RMSEs of IV are similar for the two methods, ZI-DCAE performed better with respect to the PRMSE, PMAE, and RMSE of SD overall. This implies that the clamped tanh activation function can predict more exact values in the missing interval, as intended. Moreover, the sigmoid activation function cannot generate exact zero values and cannot reflect a zero-inflated distribution. We hope that our study will inspire other researchers to apply the deep learning approach.
