# Supplementary material for: Deep Learning Approach for Imputation of Missing Values in Actigraphy Data: Algorithm Development Study
Source: JMIR Mhealth Uhealth. 2020 Jul 23;8(7):e16113. doi: 10.2196/16113 (PMC7413283; doi:10.2196/16113)
Supplement: Multimedia Appendix 8 [file mhealth_v8i7e16113_app8.docx]

# **Multimedia Appendix 8.** The accuracy of restoring Moderate-to-Vigorous Physical Activity (MVPA)

In particular, zero-inflated denoising convolutional autoencoder shows better performance in restoring 20 minutes or more of moderate-to-vigorous physical activity in NHANES data. The mean imputation and zero-inflated Poisson regression methods tend to underestimate moderate-to-vigorous physical activity in all data sets. On the validation data set, Bayesian regression is able to restore 10 minutes of moderate-to-vigorous physical activity well, but zero-inflated denoising convolutional autoencoder outperforms in restoring 30 minutes of moderate-to-vigorous physical activity. zero-inflated denoising convolutional autoencoder will be more useful for imputing missing data in cohorts that usually perform highly intensive activities.

**Table S8.** Accuracy of restoring Moderate-to-Vigorous Physical Activity (MVPA)

| Dataset | MVPA^a^  (min) | ZI-DCAE (%) | Mean  Imputation (%) | ZIP  Regression (%) | Bayesian  Regression (%) |
| --- | --- | --- | --- | --- | --- |
| NHANES^b^ |  |  |  |  |  |
|  | 0 | 93.14 | 100.00 | 95.33 | 99.71 |
|  | 10 | 10.34 | 0.0 | 25.28 | 0.00 |
|  | 20 | 11.76 | 0.0 | 5.88 | 0.00 |
|  | 30 | 84.21 | 0.0 | 73.68 | 0.00 |
| KNHANES^b^ |  |  |  |  |  |
|  | 0 | 96.97 | 100.0 | 97.16 | 99.69 |
|  | 10 | 5.40 | 0.00 | 16.21 | 0.00 |
|  | 20 | 3.44 | 0.00 | 6.89 | 0.00 |
|  | 30 | 63.63 | 0.00 | 9.09 | 0.00 |
| KCCDB^c^ |  |  |  |  |  |
|  | 0 | 95.73 | 100.00 | 99.40 | 99.70 |
|  | 10 | 3.64 | 0.00 | 8.10 | 0.00 |
|  | 20 | 0.00 | 0.00 | 0.00 | 0.00 |
|  | 30 | 38.46 | 0.00 | 7.69 | 0.00 |

^a^Duration of Moderate-to-Vigorous Physical Activiy

^b^Cutoff of MVPA was set as 1,267 by Leenders cutoff

^c^Cutoff of MVPA was set as 2,691 by Freedson VM cutoff
