# Supplementary material for: Deep Learning Approach for Imputation of Missing Values in Actigraphy Data: Algorithm Development Study
Source: JMIR Mhealth Uhealth. 2020 Jul 23;8(7):e16113. doi: 10.2196/16113 (PMC7413283; doi:10.2196/16113)
Supplement: Multimedia Appendix 9 [file mhealth_v8i7e16113_app9.docx]

# **Multimedia Appendix 9.** The result of Gaussian process imputation

**Table S9-1.** Result of Gaussian process imputation with the RBF kernel

| Measurement | NHANES^a^ | KNHANES | KCCDB |
| --- | --- | --- | --- |
| Mean of imputed data^b^ (cpm) | 0.0 | 0.0 | 0.0 |
| SD of imputed data^c^ (cpm) | 0.0 | 0.0 | 0.0 |

^a^NHANES test dataset

^b^Mean average value for each imputed data

^c^Mean standard deviation for each imputed data

**
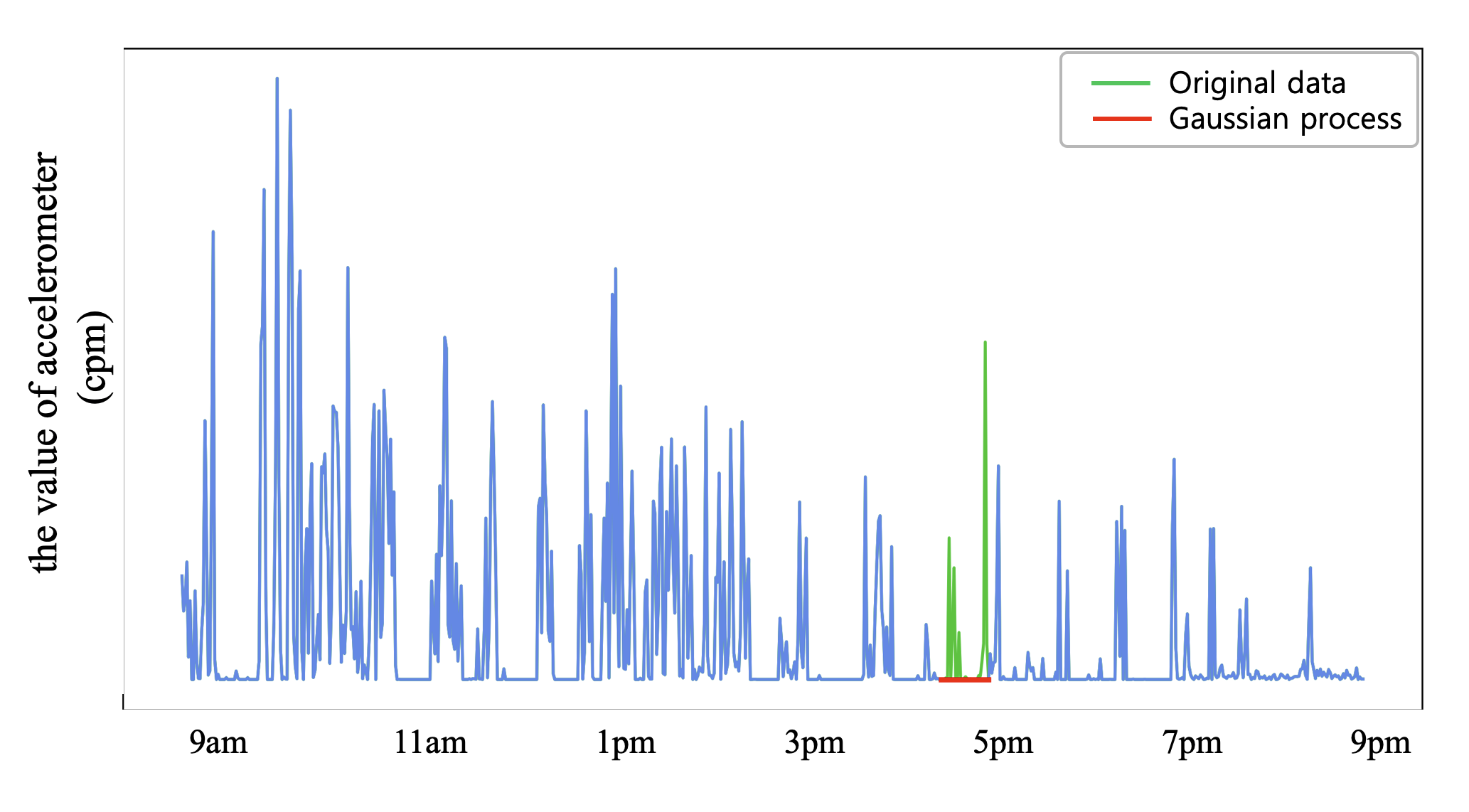
**

**
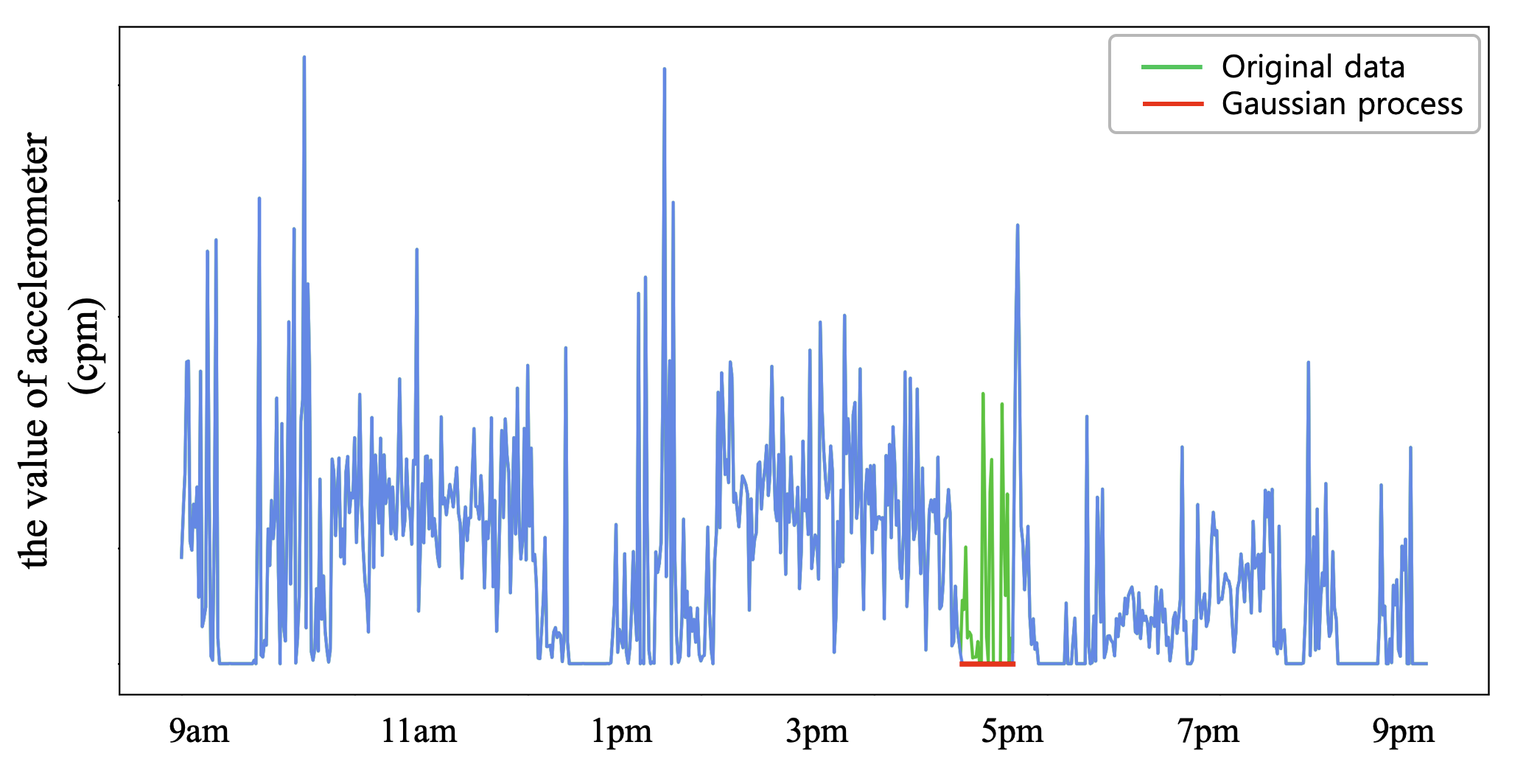
**

**Figure S9.** Example of Gaussian process imputation with the RBF kernel

Gaussian Process (GP) regression was also considered for imputing accelerometer data. However, GP regression predicted only zero values as imputed values. It seems that the zero-inflated distribution of the accelerometer makes the multivariate Gaussian distribution set a zero-mean, from which the GP infers values. Therefore, we could not use GP regression to impute actigraphy data
